# The response of PKD1L3 / PKD2L1 to acid stimuli is inhibited by capsaicin and its pungent analogs

**DOI:** 10.1111/j.1742-4658.2012.08566.x

**Published:** 2012-05

**Authors:** Sho Ishii, Azusa Kurokawa, Mikiya Kishi, Keigo Yamagami, Shinji Okada, Yoshiro Ishimaru, Takumi Misaka

**Affiliations:** 1 Central Research Institute, Mizkan Group Co.Handa, Aichi, Japan; 2 Department of Applied Biological Chemistry, Graduate School of Agricultural and Life SciencesThe University of Tokyo, Japan

**Keywords:** acid, calcium imaging, capsaicin, PKD1L3/PKD2L1, transient receptor potential channel

## Abstract

Polycystic kidney disease (PKD) 2L1 protein is a member of the transient receptor potential (TRP) ion channel family. In circumvallate and foliate papillae, PKD2L1 is coexpressed with PKD1L3. PKD2L1 and PKD1L3 interact through their transmembrane domain and the resulting heteromer PKD1L3/PKD2L1 owns a unique channel property called ‘off-responses’ to acid stimulation, although PKD2L1 does not own this property by itself. To define the pharmacological properties of the PKD1L3/PKD2L1 channel, we developed a new method to effectively evaluate channel activity using human embryonic kidney 293T cells in which the channel was heterologously expressed. This method was applied to screen substances that potentially regulate it. We found that capsaicin and its analogs, which are TRPV1 agonists, inhibited the response to acid stimuli and that the capsaicin inhibition was reversible with an IC_50_ of 32.5 μm. Capsaicin and its analogs are thus useful tools for physiological analysis of PKD1L3/PKD2L1 function.

**Database:**

Nucleotide sequence data are available in the GenBank database under the accession numbers hTRPA1, BC148423 and hTRPV3, BC104866.

## Introduction

Polycystic kidney disease (PKD) 2L1 [previously called polycystin-L or transient receptor potential (TRP) polycystin 3 (TRPP3) but renamed TRPP2] is a member of the TRP superfamily of nonselective cation channels [1,2]. According to recent reports, PKD2L1 is expressed in taste tissue, testis and a specific subset of neurons surrounding the central canal of spinal cord [3,4]. In taste tissue, PKD2L1 is expressed in a subset of taste cells, distributed in some gustatory areas named circumvallate papillae, foliate papillae, fungiform papillae and palate where ingested taste compounds are first detected. In circumvallate and foliate papillae, PKD2L1 is coexpressed and interacts with PKD1L3, and this interaction is required for its normal subcellular localization [5]. In fungiform papillae and palate, PKD2L1 is not coexpressed with PKD1L3 [3,4]. As well as its ortholog PKD2, the channel properties of PKD2L1 are altered depending on its interaction partner molecules [6]. *In vitro* analysis using human embryonic kidney (HEK) cells showed that PKD2L1 is activated by alkalization [7]. HEK cells coexpressing PKD2L1 and PKD1 showed responses to hypo-osmotic stimulation [8]. When PKD2L1 and PKD1L3 are coexpressed in HEK cells, they interact with each other through their transmembrane domain, and the resulting heteromer (PKD1L3/PKD2L1) obtains a unique channel property called ‘off-responses’ to acid stimulation. This indicates that the PKD1L3/PKD2L1 channel is gated and opens only after the removal of an acid stimulus, although the initial acid exposure is essential [9]. Off-responses to an acid stimulus were clearly observed in isolated taste cells from the circumvallate papillae, but not in those from fungiform papillae. Therefore, PKD1L3/PKD2L1 appears to generate acid evoked off-responses in taste cells [10]. Recently, knockout mice lacking PKD1L3 and/or PKD2L1 were generated and subjected to electrophysiological and behavioral analysis [11,12]. In mice lacking the PKD2L1 gene, the acid responses of fungiform taste cells and chorda tympani nerve were partially reduced compared with those in wild-type mice [12]. These results suggested that PKD2L1 in fungiform papillae partly contributes to sour taste responses. However, the functional roles of PKD1L3/PKD2L1 in circumvallate and foliate papillae have not been fully clarified yet.

For the study of TRP channels, pharmacological tools are desired to reveal physiological functions of the channels [13]. Genetic strategies and interfering RNA techniques are not able to replace the usefulness of blockers and antagonists [2]. Pharmacological tools of some TRP channels (TRPV1 and TRPM8) have been well studied. Cell-based assays using cultured cells, which heterologously express each TRP channel, enable us to screen the effective modulators for TRP channels [14,15]. However, a cell-based effective screening system for PKD1L3/PKD2L1 has not been constructed yet, since it is difficult to control pH values of the extracellular solution (acidification followed by removal of acid) to induce PKD1L3/PKD2L1 off-responses in small assay wells, where a perfusion device could not be used to apply and remove acid. Actually, there have been few reports regarding the pharmacological properties of PKD1L3/PKD2L1. An acid stimulus followed by recovery to a neutral condition is the only stimulus that activates PKD1L3/PKD2L1 [3], and effective activators other than acids and their modulators have not been identified.

TRP ion channels, some of which function as receptors with polymodal activation properties, are involved in a variety of sensory processes. For example, TRPV1 is activated by heat (>43 °C), acid, capsaicin, 2-aminoethyl diphenyl borate (2-APB) and camphor [16,17]. However, one stimulus may activate multiple TRP channels. In fact, camphor activates both TRPV3 and TRPV1 [18,19], and icilin activates both TRPA1 and TRPM8 [20,21]. Furthermore, several reports have shown that a single stimulus can have the opposite effect on multiple distinct TRP channels. Menthol activates TRPM8 but inhibits TRPA1 [22]. Cinnamaldehyde (CALD) activates TRPA1 but inhibits TRPM8 [22]. Therefore, we hypothesized that some agonists and inhibitors of other TRP channels would modulate PKD1L3/PKD2L1 activity. In this study, we used Ca^2+^ imaging analyses based on a novel neutralization method that we developed to evaluate the effects of known agonists and inhibitors of other TRP channels on PKD1L3/PKD2L1. Capsaicin and its analogs, which are TRPV1 agonists, inhibited the Ca^2+^ response to acid stimuli in HEK293T cells expressing PKD1L3/PKD2L1.

## Results

### Development of a novel method to evaluate PKD1L3/PKD2L1 activity by neutralization

**Fig 1:**
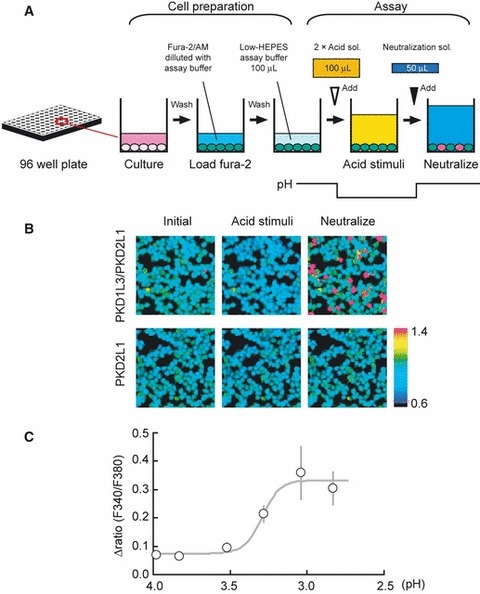
The neutralization method, a novel method for evaluating PKD1L3/PKD2L1 activity. A novel Ca^2+^ imaging method using neutralization was developed. (A) Schematic representation of the method. The cell preparation and assay steps are illustrated. In the assay step, cells are exposed to acid for 8 s and then neutralized. The lower black line schematically indicates the pH change of the extracellular solution. (B) Representative ratiometric images of Fura-2 loaded cells at the initiation of the assay, during acid stimulus and after neutralization. Upper panels indicate cells transfected with PKD1L3 and PKD2L1. Lower panels indicate cells transfected with only PKD2L1. The color scale indicates the F340/F380 ratio. Scale bar, 100 μm. (C) The pH-dependent cell response was evaluated using the neutralization method. Cells were transfected with PKD1L3 and PKD2L1. Each point represents the mean ± SEM of the fluorescence ratio change in independent trials (*n* = 6). In each trial, 100 DsRed2-positive cells were analyzed.

PKD1L3 and PKD2L1 were functionally expressed in HEK293T cells, which generated an off-response to acid stimuli [3,9,23,24]. Here, we developed a novel method for Ca^2+^ imaging using this channel. To produce a method for obtaining efficient measurements and a high-throughput screening assay, we seeded transfected cells in 96-well plates and added an acidic solution followed by a neutralization solution (Fig. 1A). This method enabled us to change the extracellular solution from acidic to neutral instead of perfusing the extracellular solution to adjust the pH. Note that with this method the pH value after neutralization was higher than 5.0, which is sufficient to activate PKD1L3/PKD2L1 after acidification [9].

The transfected cells that were seeded in 96-well plates were loaded with Fura-2/AM and rinsed with a low-HEPES assay buffer (pH 7.4) to easily change the pH value of the buffer. After stimulation with a 2.5 mm citric acid solution (pH 3.0), we added the neutralization buffer. A population of the cells that were transfected with both PKD1L3 and PKD2L1 clearly responded after neutralization, whereas few responses were observed after the acidic solution was added (Fig. 1B). As with the perfusion method, the cells transfected with PKD2L1 alone barely responded to both the acidic stimulation and neutralization, as reported previously [3,23].

We examined the relationship between the pH value of the stimulation acid and the cell response after neutralization. When various citric acid solutions at pH 2.8–4.0 were used, the cellular response after neutralization depended on the pH value of the acid solution with an EC_50_ value of pH 3.3 (Fig. 1C), which is slightly higher than that previously reported [3]. These results strongly indicate that this method is capable of detecting the channel activity of PKD1L3/PKD2L1, which shows an off-response to acidic stimuli and is applicable in rapid screening for PKD1L3/PKD2L1 modulators.

### The regulatory effect of other TRP channel agonists on PKD1L3 / PKD2L1 activity

**Fig 2:**
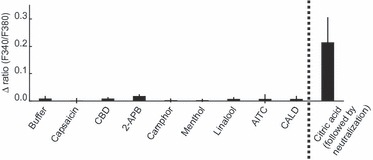
The agonistic activity of other TRP channel agonists on PKD1L3/PKD2L1. HEK293T cells transfected with PKD1L3 and PKD2L1 were subjected to Ca^2+^ imaging. Transfection and Fura-2 loading were performed prior to utilizing the neutralization method. After Fura-2 loading, cells were washed and incubated with assay buffer in 96-well plates. Each of the TRP channel agonists was diluted with assay buffer and applied to the cells. The final concentration of each compound was 100 μm. Note that acidification and neutralization were *not* performed. Each bar represents the mean ± SEM of the fluorescence ratio change from independent trials (*n* = 3–6). In each trial, 100 DsRed2-positive cells were analyzed. The rightmost bar represents the cellular response to citric acid (pH 3.0) followed by neutralization. To confirm cell responsiveness, this test was performed using the neutralization method under the same experimental conditions.

We next evaluated the regulatory effect of agonists for other TRP channels on PKD1L3/PKD2L1 activity. For this purpose, we selected the following eight TRP channel agonists: capsaicin from chili peppers for TRPV1 [16]; (−)cannabidiol (CBD), a non-psychotropic constituent of cannabis for TRPV2 [25]; camphor, used in a variety of topical analgesics and decongestants, for TRPV3 and TRPV1 [18,19]; synthetic 2-APB for TRPV1, TRPV2 and TRPV3 [26]; menthol from mint as well as linalool from lavender for TRPM8 [15,27]; and allyl isothiocyanate (AITC) from mustard oil as well as CALD from cinnamon for TRPA1 [28,29]. HEK cells expressing PKD1L3/PKD2L1 barely responded to each agonist when it was applied alone (Fig. 2). Therefore these compounds had only a slight ability to activate PKD1L3/PKD2L1.

**Fig 3:**
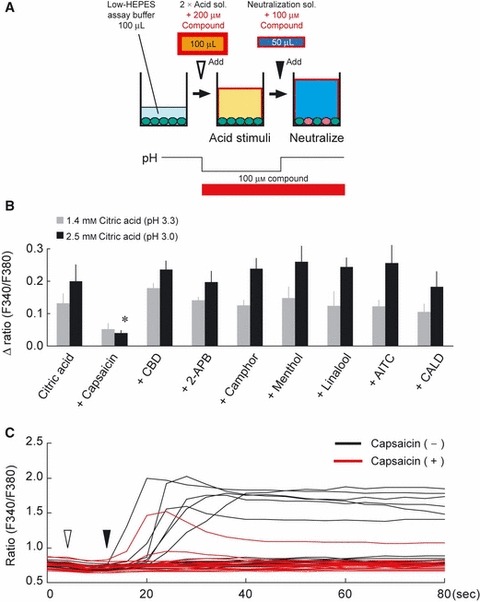
The regulatory effect of other TRP channel agonists on PKD1L3/PKD2L1. HEK293T cells transfected with PKD1L3 and PKD2L1 were subjected to Ca^2+^ imaging using the neutralization method. (A) Schematic representation of the experiments. The lower black line schematically indicates the pH change of the extracellular solution. The horizontal red bar indicates the presence of a test compound. (B) In the presence of 100 μm of the TRP channel agonists, cellular responses to a 1.4 mm (gray bar) and 2.5 mm (black bar) citric acid solution followed by neutralization were evaluated. Each bar represents the mean ± SEM of the fluorescence ratio change from independent trials (*n* = 5). In each trial, 100 DsRed2-positive cells were analyzed. The pH values of the 1.4 mm and 2.5 mm citric acid solutions were 3.3 and 3.0, respectively, regardless of the absence or presence of the test compounds in almost all cases. As an exception, the 2.5 mm citric acid solution containing 100 μm 2-APB had a pH of 3.1. The significance of the difference between the control (citric acid) and test values was determined using a one-way analysis of variance (ANOVA) followed by Dunnett’s test. *P < 0.05. (C) Sequential measurement of the F340/F380 ratiometric values. Black lines reflect 20 representative cells stimulated by 2.5 mm citric acid (pH 3.0). Red lines reflect 20 representative cells stimulated by 2.5 mm citric acid in the presence of 100 μm capsaicin (pH 3.0). The white and black arrowheads indicate the time points of the acid stimulus and the neutralization, respectively.

PKD1L3/PKD2L1 expressing HEK cells were stimulated by the application of a citric acid solution (pH 3.0 or 3.3) containing the TRP channel agonists (final concentration 100 μm). The cell response was examined after the addition of neutralizing solutions containing 100 μm of each agonist (Fig. 3A). In the absence of TRP channel agonists, cells showed a 0.13 ± 0.03 and 0.20 ± 0.05 increase in fluorescence ratio after stimulation with 1.4 mm citric acid (pH 3.3) or 2.5 mm citric acid (pH 3.0), respectively, followed by neutralization (Fig. 3B). Note that in the presence of 100 μm capsaicin the fluorescence ratio change after neutralization was significantly smaller than that in the absence of capsaicin at pH 3.0 (Fig. 3B). No significant difference was observed in the fluorescence ratio between the absence and presence of CBD, 2-APB, camphor, menthol, linalool, AITC or CALD under any of the pH conditions (Fig. 3B).

We also examined a time course for the channel activity. A subset of the transfected cells showed an increase in F340/F380 ratiometric values only after neutralization following acidification. These increased ratiometric values were sustained for more than 60 s (Fig. 3C). However, in the presence of capsaicin, only a few cells showed an increase in the fluorescence ratio (Fig. 3C). Our results strongly indicate an inhibitory activity for capsaicin on the PKD1L3/PKD2L1 channel, and thus the effect of capsaicin was analyzed in more detail.

### The concentration-dependent inhibitory effect of capsaicin on PKD1L3/PKD2L1

**Fig 4:**
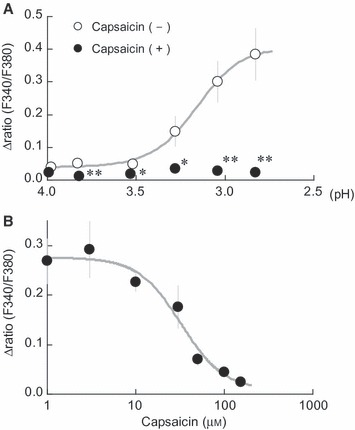
Concentration-dependent inhibitory effect of capsaicin on PKD1L3/PKD2L1. HEK293T cells transfected with PKD1L3 and PKD2L1 were subjected to Ca^2+^ imaging using the neutralization method. (A) The pH dependence of PKD1L3/PKD2L1 activation in either the presence (closed circle) or absence (open circle) of 100 μm capsaicin. Each point represents the mean ± SEM of the fluorescence ratio change from independent trials (*n* = 5). In each trial, 100 DsRed2-positive cells were analyzed. The significance for the differences between the absence and presence of capsaicin at each pH was determined using a paired *t* test; *P < 0.05, **P < 0.01. (B) Concentration dependence of the inhibitory effect of capsaicin. Cells were stimulated with 2.5 mm citric acid (pH 3.0) followed by neutralization in the presence of capsaicin. Each point represents the mean ± SEM of the fluorescence ratio change from independent trials (*n* = 4). In each trial, 100 DsRed2-positive cells were analyzed.

The pH dependence of the PKD1L3/PKD2L1 response was evaluated in the absence and presence of capsaicin (Fig. 4A). HEK293T cells transfected with both PKD1L3 and PKD2L1 were seeded in 96-well plates and subjected to Ca^2+^ imaging. In the absence of capsaicin, the cell responses after neutralization increased in a pH-dependent manner with acid stimulation, as described above (Fig. 1C). In contrast, in the presence of 100 μm capsaicin, cells barely responded to stimulation by acid. The fluorescence ratio increase that followed stimulation with pH 3.8, 3.5, 3.3, 3.0 and 2.8 was significantly smaller than that in the absence of capsaicin (Fig. 4A).

The response of cells transfected with PKD1L3 and PKD2L1 to an acidic solution (pH 3.0) was evaluated in the presence of various concentrations (1, 3, 10, 30, 50, 100 and 150 μm) of capsaicin. We maintained capsaicin at the indicated concentrations both during the acid stimulation and after neutralization by adding it to the acid and neutralization solutions. The fluorescence ratio change decreased in a concentration-dependent manner when capsaicin was present (Fig. 4B), and the concentration-dependent inhibitory curve had an IC_50_ value of 32.5 ± 10.2 μm. In the presence of 150 μm capsaicin, the response was almost completely eliminated.

### The specificity of the inhibitory effect of capsaicin

**Fig 5:**
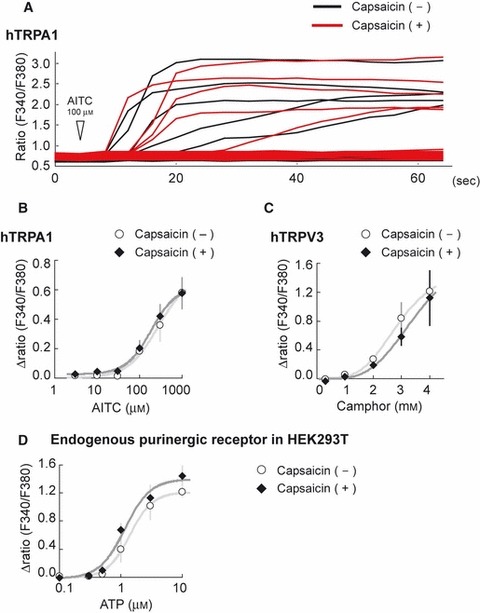
The effect of capsaicin on the Ca^2+^ response in HEK293T cells mediated by other TRP channels or by the endogenous receptor. HEK293T cells transiently transfected with either hTRPA1 or hTRPV3 and non-transfected HEK293T cells, which endogenously express a purinergic receptor, were subjected to Ca^2+^ imaging in either the presence or the absence of capsaicin. Stimulations were performed via the application of the cognate ligands (AITC, camphor and ATP). (A) Sequential measurement of the F340/F380 ratiometric values in hTRPA1-expressing cells. Black lines represent 20 representative cells stimulated by 100 μm AITC. Red lines represent 20 representative cells stimulated by 100 μm AITC in the presence of 100 μm capsaicin. The white arrowhead indicates the time point for the AITC stimuli. The concentration dependence of the cell responses mediated by (B) hTRPA1, (C) hTRPV3. (D) The endogenous purinergic receptor. Ca^2+^ imaging was performed in either the presence (closed square) or the absence (open circle) of 100 μm capsaicin. Each point represents the mean ± SEM of the fluorescence ratio change from independent trials (*n* = 3). In each trial, 100 DsRed2-positive cells were analyzed. The expression vectors for hTRPA1 and hTRPV3 were generated by subcloning the coding regions (BC148423 and BC104866) into pcDNA5/FRT (Invitrogen) and pEAK10 (EdgeBio Systems, Gaithersburg, MD, USA) vectors, respectively.

To confirm the specificity of the inhibitory activity of capsaicin, we also examined the effect of capsaicin on the Ca^2+^ responses of HEK293T cells mediated by other TRP channels. The time course for the Ca^2+^ response in HEK293T cells heterologously transfected with hTRPA1 was evaluated in either the absence or the presence of 100 μm capsaicin. hTRPA1 was activated by the application of its cognate ligand (100 μm AITC). When 100 μm AITC was applied in the presence of 100 μm capsaicin, a clear response was observed via Ca^2+^ imaging analysis that was similar to the result obtained without 100 μm capsaicin (Fig. 5A). Furthermore, with respect to concentration dependence in the cells, the responses to AITC stimuli were comparable in the absence and presence of 100 μm capsaicin (Fig. 5B). Additionally, the Ca^2+^ response in HEK293T cells mediated by heterologously expressed hTRPV3 and the response of endogenous purinergic receptors [30] to their ligands (camphor and ATP, respectively) in the presence of 100 μm capsaicin were clearly observed, as with the absence of capsaicin (Fig. 5C,D). These results indicate that capsaicin does not non-specifically inhibit Ca^2+^ responses in HEK293T cells and that the cells did not lose responsivity in the presence of a high capsaicin concentration. These results also indicate that capsaicin is an inhibitor of the PKD1L3/PKD2L1 channel.

### Evaluation of the inhibitory activity of capsaicin analogs on PKD1L3/PKD2L1

**Fig 6:**
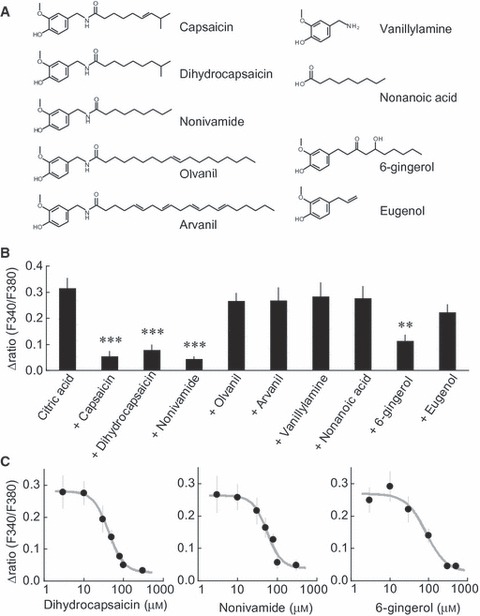
The inhibitory effect of capsaicin analogs on PKD1L3/PKD2L1. HEK293T cells transfected with PKD1L3 and PKD2L1 were subjected to Ca^2+^ imaging using the neutralization method. (A) Formulae for the capsaicin analogs. (B) In the presence of 100 μm of the capsaicin analog, cell responses to a 2.5 mm citric acid solution followed by neutralization were evaluated. Each bar represents the mean ± SEM of the fluorescence ratio change from independent trials (*n* = 11). In each trial, 100 DsRed2-positive cells were analyzed. The pH value of the 2.5 mm citric acid solution was 3.0, regardless of the absence or presence of the test compounds in all cases. The significance for the differences between the control (citric acid) and test values was determined using ANOVA followed by Dunnett’s test. **P < 0.01, ***P < 0.001. (C) Concentration dependence of the inhibitory effect of capsaicin analogs. Cells were stimulated with 2.5 mm citric acid (pH 3.0) followed by neutralization in the presence of a capsaicin analog. Each point represents the mean ± SEM of the fluorescence ratio change from independent trials (*n* = 10). In each trial, 100 DsRed2-positive cells were analyzed.

We evaluated the inhibitory effect of capsaicin analogs on PKD1L3/PKD2L1 activity to elucidate the structural properties required for the inhibition. Because capsaicin is an acid amide generated from vanillylamine and 8-methyl-6-nonen acid (Fig. 6A), we first examined the contribution of acyl-chain-composing acid amides. Dihydrocapsaicin, nonivamide, olvanil and arvanil are acid amides of vanillylamines whose acyl chains are constituted of 8-methyl-nonanoic acid, nonanoic acid, oleic acid and arachidonic acid, respectively (Fig. 6A). In the presence of 100 μm dihydrocapsaicin or nonivamide, the fluorescence ratio change was significantly smaller than that in the absence of each compound, and olvanil and arvanil had only a small effect on cellular responses (Fig. 6B). However, vanillylamine and nonanoic acid, which are constituent elements of capsaicin and nonivamide, showed no inhibitory effect (Fig. 6B). The compounds 6-gingerol and eugenol contain the vanillyl group and acyl chain in addition to capsaicin, but they do not have an amide moiety in their chemical structures (Fig. 6A). In the presence of 6-gingerol, the fluorescence ratio change after acid stimuli was slightly but significantly smaller. Eugenol did not significantly inhibit cellular responses (Fig. 6B).

**Fig 7:**
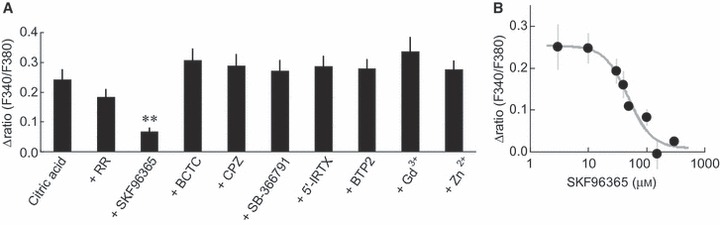
The inhibitory effect of TRP channel inhibitors on PKD1L3/PKD2L1. HEK293T cells transfected with PKD1L3 and PKD2L1 were subjected to Ca^2+^ imaging using the neutralization method. (A) In the presence of TRP channel inhibitors, cellular responses to 2.5 mm citric acid solution followed by neutralization were evaluated. Each bar represents the mean ± SEM of the fluorescence ratio change from independent trials (*n* = 6). In each trial, 100 DsRed2-positive cells were analyzed. Ruthenium red (RR), SKF96365, capsazepine (CPZ) and Gd^3+^ were applied at 100 μm concentration. BCTC, SB-366791, 5′-IRTX and BTP2 were applied at 10 μm concentration. ZnCl_2_ was applied at 1 mm concentration. The pH value of the 2.5 mm citric acid solution was 3.0, regardless of the absence or presence of the test compounds in almost all cases. As an exception, the pH of the 2.5 mm citric acid solution containing 100 μm RR was 3.1. The significance of the differences between the control (citric acid) and the test values was determined using ANOVA followed by Dunnett’s test. **P < 0.01. (B) Concentration dependence of the inhibitory effect of SKF96365. Cells were stimulated with 2.5 mm citric acid (pH 3.0) followed by neutralization in the presence of SKF96365. Each point represents the mean ± SEM of the fluorescence ratio change from independent trials (*n* = 5). In each trial, 100 DsRed2-positive cells were analyzed.

Concentration dependence was evaluated for the compounds showing inhibitory activity (dihydrocapsaicin, nonivamide and 6-gingerol). In all cases, the fluorescence ratio changes decreased in a concentration-dependent manner. The concentration-dependent inhibitory curves had an IC_50_ value of 55.0 ± 14.2 μm for dihydrocapsaicin and 42.2 ± 8.9 μm for nonivamide. These values are comparable to those obtained for capsaicin (Figs 4B, 6C). However, the IC_50_ of 6-gingerol was 85.9 ± 34.7 μm, which is approximately 2.5-fold higher than that of capsaicin (Figs 4B, 6C). The inhibitory potency is ranked as follows: capsaicin ∼ dihydrocapsaicin ∼ nonivamide > 6-gingerol. We also evaluated the effect of general TRP channel inhibitors [17]. SKF96365, a broad TRP channel pore blocker, had an inhibitory effect on PKD1L3/PKD2L1 with an IC_50_ value of 48.1 ± 10.8 μm (Fig. 7). The IC_50_ values of capsaicin, dihydrocapsaicin and nonivamide are comparable with that of the well-known TRP channel pore blocker, SKF96365.

### The reversibility of the inhibitory effect of capsaicin on PKD1L3/PKD2L1

**Fig 8:**
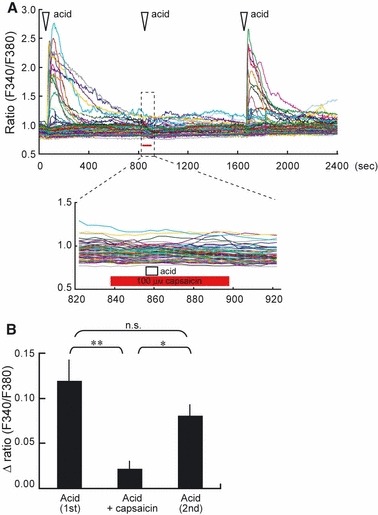
The reversibility of the inhibitory effect of capsaicin on PKD1L3/PKD2L1. HEK293T cells transfected with PKD1L3 and PKD2L1 were subjected to Ca^2+^ imaging using the perfusion method. (A) Sequential measurement of the F340/F380 ratiometric values. Each line reflects 100 representative cells. In the upper panel, the arrowheads indicate the time point for the 25 mm citric acid (pH 2.8) stimulation applied for 6 s. The horizontal red bar indicates the presence of 100 μm capsaicin. The lower panel is a magnified figure of the region surrounded by the dashed line in the upper panel. The horizontal white and red bars indicate the time point for acid application and the presence of 100 μm capsaicin, respectively. (B) The fluorescence ratio change defined in (A). Each bar represents the mean ± SEM of the fluorescence ratio change from independent trials (*n* = 6). In each trial, 100 DsRed2-positive cells were analyzed. The significance of the differences was determined using ANOVA followed by Tukey’s test. *P < 0.05, **P < 0.01; n.s., not significant.

Capsaicin is probably an inhibitor of PKD1L3/PKD2L1, as described above. To examine the reversibility of this effect, we exposed HEK293T cells transfected with PKD1L3 and PKD2L1 to a constant flow of the assay buffer (approximately 10 mL·min^−1^) using a perfusion method and performed a sequential stimulation. First, 25 mm citric acid diluted in assay buffer (pH 2.8) was applied to cells for 6 s, and the cells were subsequently washed with the assay buffer (pH 7.4). After being washed, some cells showed increased F340/F380 ratiometric values. When the ratiometric values recovered to near baseline (after 800 s), a subsequent acid application (pH 2.8) was performed in the presence of 100 μm capsaicin, and the cells were washed with the assay buffer containing 100 μm capsaicin. Only a few cells showed increased ratiometric values. After the capsaicin was removed, the fluorescence ratio changes in responses to the acid stimuli were significantly higher than those in the presence of capsaicin, although they were slightly smaller than those of the first responses (Fig. 8). These results indicate that the inhibitory effect of capsaicin is partially reversible.

### In mouse taste cells, responses to acid are inhibited by capsaicin

**Fig 9:**
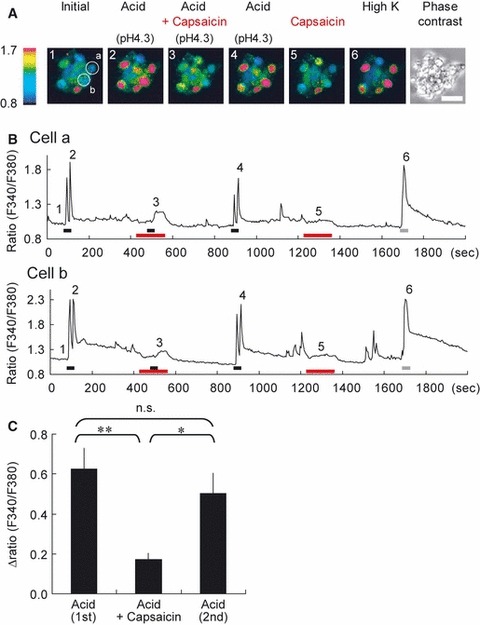
The effect of capsaicin on mice circumvallate taste cell responses to acidic stimuli. Taste cells were isolated from the circumvallate papillae of C57BL/6J male mice, and the Ca^2+^ response to acid stimulation in isolated cells loaded with Fura-2 was monitored. (A) Representative F340/F380 ratiometric images of circumvallate taste cells at the time points indicated in (B) as 1, 2, 3, 4, 5 and 6. The color scale indicates the F340/F380 ratio. The rightmost panel indicates the phase contrast image. Scale bar, 50 μm. (B) The F340/F380 ratiometric values of two representative cells (cells a and b) indicated in (A) as a and b. Horizontal black bars indicate the time points of the 3.0 mm citric acid (pH 4.3) stimulus. The red bars indicate the time points of the 100 μm capsaicin presence. The gray bars indicate the time points of the 50 mm High K stimulus. (C) The mean ± SEM of F340/F380 ratiometric value changes of taste cells (*n* = 10 cells from five taste buds) at acid-induced off-responses. The 10 cells that showed off-responses to acidic stimuli of the 11 High K responsive cells were analyzed. The F340/F380 ratiometric value changes were defined as the peak height of the off-response from the baseline just before each acid stimulus. The significance of the difference was determined by ANOVA followed by Tukey’s test. *P < 0.05, **P < 0.01; n.s., not significant.

**Fig 10:**
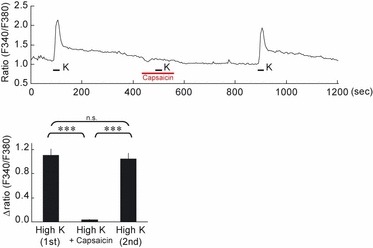
The effect of capsaicin on mice circumvallate taste cell responses to high K^+^ stimuli. Mice circumvallate taste cells were subjected to Ca^2+^ imaging. The upper panel shows the F340/F380 ratiometric values from a representative cell. The black bars indicate the time points for the 50 mm K^+^ stimulus. The red bar indicates the time points for the presence of 100 μm capsaicin. The lower panel shows the mean ± SEM of F340/F380 ratiometric value changes in the taste cells (*n* = 9 cells from five taste buds). The F340/F380 ratiometric value changes were defined as the peak height of the response from the baseline just before each high K^+^ stimulus. The significance for the difference was determined using ANOVA followed by Tukey’s test. ***P < 0.001; n.s., not significant.

A recent report examined the response of mouse circumvallate taste cells to citric acid stimulation and showed an off-response. All the responding cells were GAD67-positive cells, and most GAD67-positive cells had been shown to express the PKD2L1 protein, indicating a relationship between acid sensing and the PKD1L3/PKD2L1 channel in circumvallate taste buds [10]. GAD67 is expressed in a subset of taste cells determined as type III cells [31], and type III cells are thought to be sour sensor cells [32,33]. Here, the inhibitory effect of capsaicin on the response to acid stimulation was evaluated in mouse circumvallate taste cells by a similar procedure. Taste cells were isolated from the circumvallate papillae of C57BL/6J male mice and loaded with Fura-2. The Ca^2+^ response to acid stimulation in isolated cells was monitored. Acid stimuli were performed with 3.0 mm citric acid solution (pH 4.3). The pH value of acid solution used to stimuli taste cells was higher than that to stimuli HEK293T cells because in circumvallate taste cells off-responses were induced by acids with a pH value of < 5.0 [10]. The type III cell is known as High K sensitive [34]. Stimulation with a 50 mm KCl solution (High K) was performed to select type III cells and confirm cell viability. Eleven cells responded to the High K stimulus. Ratiometric values representative of two cells (cells a and b in Fig. 9A) are shown in Fig. 9B. A total of 10 of 11 High K responsive cells (90.9%) showed increased F340/F380 ratiometric values after the first acid stimulation. Although cells were stimulated with acid only once, two independent transient increases in the ratiometric values were observed during and after exposure to the acid (Fig. 9A,B); these corresponded to the on- and off-responses. Both responses showed a robust, sharp peak increase in the F340/F380 ratiometric value. After the ratiometric values recovered to near baseline (after 400 s), acid stimulation in the presence of 100 μm capsaicin was performed. Although off-responses were observed, the peak heights of the responses were significantly lower than those observed in the absence of capsaicin (Fig. 9A–C), and the shape of the off-response peak became broader (Fig. 9B). An unexpected decrease in the peak height of the on-response was also found in the presence of capsaicin. After the capsaicin was washed out of the cells, a second acid stimulation evoked robust on- and off-responses (Fig. 9A–C). No clear response was observed in cells exposed only to capsaicin, although ratiometric values increased slightly (Fig. 9B). These results indicate that capsaicin also inhibits mice taste cell responses to acid and that the inhibition is reversible. To confirm specificity, we performed an experiment with mouse taste cells to test the effect of capsaicin on the response to high K^+^. Unexpectedly, the taste cells’ response to high K^+^ was also reversibly inhibited in the presence of capsaicin under our experimental conditions (Fig. 10), as in the case of acid stimulation.

## Discussion

In the present study, we demonstrated that the response of cells expressing PKD1L3/PKD2L1 to acid stimulation followed by neutralization was inhibited in the presence of capsaicin, a well-known TRPV1 agonist (Figs 3 and 4). Because the effect was reversible (Fig. 8), the inhibitory effects of capsaicin were probably derived from a transient interaction between the PKD1L3/PKD2L1 proteins and capsaicin. Our findings emphasize that capsaicin has the opposite effect on TRPV1 and PKD1L3/PKD2L1, although both belong to the TRP channel family.

Some chemical compounds that are structurally related to capsaicin also show an inhibitory effect (Fig. 6). Capsaicin, dihydrocapsaicin and nonivamide inhibited cell responses with comparable IC_50_ values, whereas olvanil and arvanil did not inhibit PKD1L3/PKD2L1 (Fig. 6B,C). These results suggest that the overall size of the molecule is more important than the double bond and the methyl group on the acyl chain (Fig. 6A). Certain structural variants also inhibited PKD1L3/PKD2L1. This indicates that other compounds, including the vanillyl moiety, could act to inhibit PKD1L3/PKD2L1. Further screening may elucidate new inhibitors, and the neutralization method developed in this study could be a powerful tool for such screening (Fig. 1). This method enables us to decrease the time required for the cell assay by approximately one-fifth compared with the conventional perfusion method, and it is applicable to a cellular assay screening system with an automated assay device. In addition, the consumption of test compounds is reduced to approximately 1/50.

In mouse taste cells, capsaicin significantly inhibited the response to acidic stimuli (Fig. 9C). It was reported that off-responses were detected with a high incidence in PKD2L1-positive type III cells isolated from circumvallate papillae, but not from fungiform papillae. This result does not contradict the notion that acid-evoked off-responses in taste cells are produced by PKD2L1/PKD1L3 [10]. In our experiment, the inhibitory effect of capsaicin on PKD1L3/PKD2L1 was observed in a heterologous system (Figs 3, 4 and 8), and taste cell viability was maintained after capsaicin was washed out of the cells (Fig. 9). Therefore, the inhibition of off-responses by capsaicin in taste cells may reflect the inhibitory effect of capsaicin on PKD1L3/PKD2L1. We could not rule out the possibility that our result reflected the effect of capsaicin on secondarily activated Ca^2+^ channels, because taste cell response to high K^+^ was also reversibly inhibited in the presence of capsaicin (Fig. 10). Actually, similar results have been reported showing that voltage gated Ca^2+^ channels were inhibited by capsaicin in primary cultured DRG and TG neurons and other cell types [35–37]. Although we should await some new molecular biological and pharmacological information regarding the signal transduction in taste cells, our finding that capsaicin reversibly suppresses the taste cell response to sour stimulation provides important information indicating a characteristic of this pungent food factor.

It has been reported that peppers or foods containing 2.6–1471.5 μg capsaicin per gram of fresh weight are sold commercially in the USA [38]. When these foods are ingested, capsaicin will reach TRPV1 expressed on somatosensory nerves and evoke a pungent sensation. Additionally, capsaicin is accessible to the oral cavity in sufficient concentrations to inhibit PKD1L3/PKD2L1 activation, because the IC_50_ value of capsaicin for PKD1L3/PKD2L1 inhibition in our study was 32.5 μm, which roughly corresponds to 9.9 μg capsaicin per gram (Fig. 4B). Several reports have shown that the intensity of the taste of acid is decreased by capsaicin or capsicum oleoresin treatment [39,40]. Our data also reveal potential mechanisms of oral perception of whole foods containing both acidic and pungent compounds.

## Materials and methods

### Materials

Capsaicin, camphor, CALD, vanillylamine, nonanoic acid and 6-gingerol were obtained from Wako Pure Chemical (Osaka, Japan). CBD was obtained from Tocris Bioscience (Ellisville, MO, USA). The chemicals 2-APB, dihydrocapsaicin, olvanil and arvanil were obtained from Cayman Chemical (Ann Arbor, MI, USA). l-Menthol was obtained from Kanto Chemical (Tokyo, Japan). Linalool was obtained from BASF (Ludwigshafen, Germany). AITC and eugenol were obtained from Tokyo Chemical Industry (Tokyo, Japan). Nonivamide was obtained from Enzo Life Sciences (Plymouth Meeting, PA, USA). As stock solutions, capsaicin, CBD, 2-APB, camphor, l-menthol, linalool, AITC, CALD, dihydrocapsaicin, nonivamide, vanillylamine, nonanoic acid, 6-gingerol and eugenol were dissolved in dimethyl sulfoxide. Olvanil and arvanil were dissolved in ethanol.

### Sample solutions

The assay buffer used for Ca^2+^ imaging was composed of 10 mm HEPES, 130 mm NaCl, 10 mm glucose, 5 mm KCl, 2 mm CaCl_2_ and 1.2 mm MgCl_2_ (pH adjusted to 7.4 using NaOH). In another assay buffer (a low-HEPES assay buffer) for Ca^2+^ imaging, the concentration of HEPES was reduced to 1 mm and the concentrations of the other components were identical to those in the assay buffer described above. Citric acid was diluted with the assay buffer to produce acidic solutions of pH 4.3 and 2.8 (corresponding to 3 and 25 mm citric acid, respectively) and with the low-HEPES assay buffer to produce acidic solutions of pH 4.0, 3.8, 3.5, 3.3, 3.0 and 2.8 (corresponding to 0.45, 0.55, 0.9, 1.4, 2.5 and 4.0 mm citric acid, respectively). The neutralization solution was composed of 100 mm HEPES, 80 mm NaOH, 130 mm NaCl, 10 mm glucose, 5 mm KCl, 2 mm CaCl_2_ and 1.2 mm MgCl_2_ (pH 8.2). Stock solutions of each reagent were diluted with assay buffer, acid solution or neutralization solution at the desired concentrations. In all experiments, the final concentration of dimethyl sulfoxide was < 1.0% (v/v) and the concentration of ethanol was < 0.1% (v/v).

### Cell culture and transfection

HEK293T cells were cultured at 37 °C in DMEM (Sigma-Aldrich Japan, Tokyo, Japan) supplemented with 10% FBS (Invitrogen, Carlsbad, CA, USA). Cells were seeded onto 35-mm dishes and transiently transfected with the expression vectors using the Lipofectamine 2000 reagent (Invitrogen). The expression vectors for mouse PKD1L3 and PKD2L1 were generated by subcloning the coding regions into the pDisplay (Invitrogen) and pCI (Promega, Madison, WI, USA) vectors, respectively, as described previously [3]. The vectors for PKD1L3, PKD2L1 and red fluorescent protein (DsRed2, pDsRed2-N1; Takara Bio Inc., Shiga, Japan) were transfected at a ratio of 10: 10: 0.4.

### Ca^2+^ imaging

For the neutralization assay, cells were transferred to Lumox multiwell 96-well plates (Sarstedt, Numbrecht, Germany) 6 h after transfection. The cells were incubated for an additional 24–28 h, washed with assay buffer, and loaded with 5 μm Fura-2/AM (Invitrogen) for 30 min at room temperature. The cells were rinsed with the low-HEPES assay buffer and incubated in 100 μL of the low-HEPES buffer for at least 10 min. Ligand application was performed by adding 100 μL of the 2× acid solution diluted with low-HEPES assay buffer. To neutralize the buffer, we added 50 μL of the neutralization solution to each well 8 s after acid application and monitored the intensity of Fura-2 fluorescence. In this assay system, the final pH of the solutions converged to approximately neutral for all the stimuli we examined. For example, the citric acid solution pH values 4.0, 3.8, 3.5, 3.3, 3.0 and 2.8 were neutralized to pH 8.0, 8.0, 7.9, 7.6, 7.4 and 7.2, respectively.

For the perfusion assay, cells were seeded onto glass-base dishes (35 mm in diameter) and transiently transfected with the expression vector. These cells were washed with assay buffer 31–34 h after transfection. The cells were loaded with 5 μm Fura-2/AM for 30 min at room temperature. The cells were then rinsed and incubated in the assay buffer for at least 10 min. A perfusion device was used to apply the ligand solutions and to wash the cells. The cells were exposed to a constant flow (approximately 10 mL·min^−1^) of assay buffer, and the acidic solutions were applied for approximately 6 s. The cells were washed with the assay buffer immediately after application of an acidic solution, and the intensity of Fura-2 fluorescence was monitored.

The Fura-2 fluorescence intensity was measured at excitation wavelengths of 340 and 380 nm and at 510 nm using a computer-controlled filter exchanger (Lambda 10-2; Sutter, San Rafael, CA, USA), a MicroMax cooled charge-coupled device camera (Princeton Instruments, Trenton, NJ, USA) and an inverted fluorescence microscope (IX-70; Olympus, Tokyo, Japan). Images were recorded at 4-s intervals and analyzed using the metafluor software (Molecular Devices, Sunnyvale, CA, USA). The exposure times for each fluorescence acquisition, following excitation by the 340 and 380 nm wavelengths, were 600 and 300 ms, respectively. Changes in the intracellular calcium ion concentration are represented as changes in the ratio of the fluorescence emitted at the two excitation wavelengths (F340/F380). We randomly selected 100 DsRed2-positive cells (regarded as transfected cells) and determined the mean fluorescence ratio change for them after each stimulation. The response curves were fitted with Hill equations [41].

### Analysis of taste cell response

Animal experiments were approved by the Animal Care and Use Committees of the University of Tokyo. Adult (>8 weeks) C57BL/6J male mice were killed by cervical dislocation, and their tongues were isolated. Tyrode’s solution containing 2 mg·mL^−1^ collagenase (Sigma-Aldrich Japan) was injected under the epithelium surrounding the circumvallate papillae and incubated for 7 min. The epithelium was peeled and bathed in Tyrode’s solution containing 2 mg·mL^−1^ collagenase for 30 s and then in Ca^2+^- and Mg^2+^-free Tyrode’s solution for 15 min at room temperature. Taste cells were drawn into a glass capillary with gentle suction. Isolated taste cells were transferred to glass-base dishes (35 mm in diameter) coated with Cellmatrix type I-C (Nitta Gelatin Inc., Osaka, Japan). Isolated taste cells were loaded with 5 μm Fura-2/AM for 30 min at room temperature. A perfusion device was used to apply the ligand solutions and wash the cells. The cells were exposed to a constant flow (approximately 5 mL·min^−1^) of the assay buffer. Ligand solutions were applied for approximately 20 s. The intensity of Fura-2 fluorescence was monitored as described previously except in terms of the exposure times. The exposure times of each fluorescence acquisition using wavelength excitations of 340 and 380 nm were 1500 and 750 ms, respectively.

## Abbreviations

AITC: allyl isothiocyanate
2-APB: 2-aminoethyl diphenyl borate
CALD: cinnamaldehyde
CBD: (–)cannabidiol
HEK: human embryonic kidney
PKD: polycystic kidney disease
TRP: transient receptor potential
